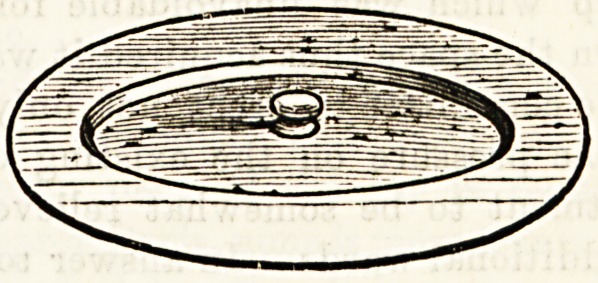# Practical Departments

**Published:** 1894-07-07

**Authors:** 


### PRACTICAL DEPARTMENTS.

### COMBINED BED-TABLE AND REST.

Dr. Stocker, Weston House, Richmond Gardens, Forest Gate, E., has brought to our notice an original design of his for a bed-table, which is capable also of being used as a bed-rest. The illustrations which we give will serve better than

Bed Table in Position.

any verbal description to bring a general idea of the plan upon which the arrangement is carried out before our readers. The mechanical adjustment is very ingenious, and really requires to be seen to be properly understood.

Sketch No. 1 shows the table in position by the bed ready for use. The stand is heavily weighted, and is so constructed

Bed Table Showing Book-rest.

that it can be fixed to the side rail of the bed, thus the table when in position across the bed is absolutely steady. The second drawing gives the table with its little book rest raised, and shows, too, a small slab for the convenient disposal of a glass or cup, which pulls out on the left-hand side. Of course the height is adjustable. In the drawing the table is fixed

Bed Table Arranged as Back-rest.

at its lowest, but it may be raised as much as desired, and swings round also, according to fancy.

The third picture shows the table detached from the stand and raised by strong supports to form a back rest. Many people, of course, prefer a cane or webbing back by way of support, but to some the greater hardness of wood is no objection, and it is undeniably sometimes an advantage to be able to make two separate and distinct uses of one such appliance. When not in use the whole arrangement will fold over, and can be disposed of neatly under the bed. Dr. Stocker's invention has, we understand, only been carried out experimentally so far, but the specimen submitted for our inspection is very well made and finished, and is attractive in appearance. It will probably be too expensive a luxury for very general use, but in many private sick-rooms will be welcomed.

### A BOON FOR THE SICK ROOM.

The Sanitary Chamberine Cover, designed and patented by Mr. H. Lewis, Worley Road, St. Albans, Herts, will be of great value in the sick room, and supplies a distinct want. No chamber utensil in the sick room should be without a lid, and these covers, being absolutely unbreakable, light, clean, non-absorbent, and noiseless, are quite the best that could be used. We cordially recommend them. Price 1s. each.

## Figures and Tables

**Figure f1:**
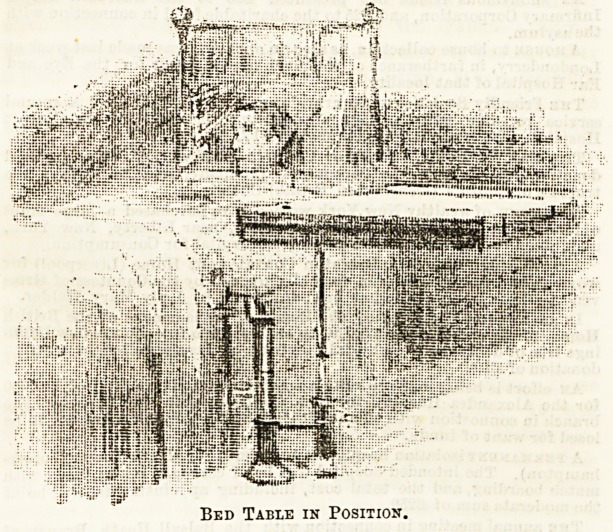


**Figure f2:**
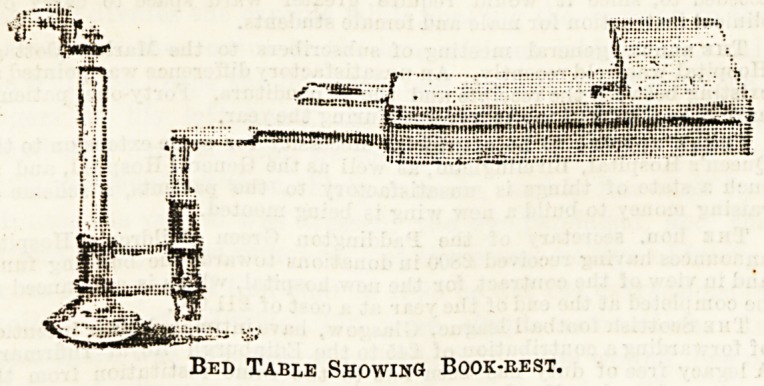


**Figure f3:**
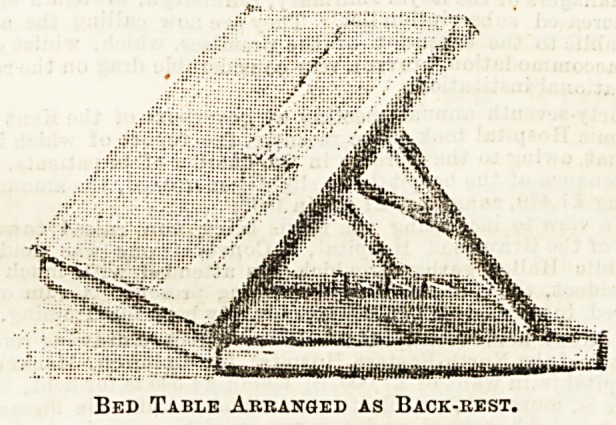


**Figure f4:**